# Current Advances in Virus-Like Particles as a Vaccination Approach against HIV Infection

**DOI:** 10.3390/vaccines4010002

**Published:** 2016-01-22

**Authors:** Chongbo Zhao, Zhujun Ao, Xiaojian Yao

**Affiliations:** 1 Laboratory of Molecular Human Retrovirology, Department of Medical Microbiology, Faculty of Medicine, University of Manitoba, Winnipeg, MB R3E 0J9, Canada; chongbozhao@yahoo.com (C.Z.); Zhujun.Ao@umanitoba.ca (Z.A.); 2 Department of Microbiology, School of Basic Medical Sciences, Central South University, Changsha 410078, Hunan, China

**Keywords:** HIV-1, virus-like paticles (VLPs), vaccine, dendritic cells-based immunotherapy, adjuvants, dendritic cells

## Abstract

HIV-1 virus-like particles (VLPs) are promising vaccine candidates against HIV-1 infection. They are capable of preserving the native conformation of HIV-1 antigens and priming CD4+ and CD8+ T cell responses efficiently via cross presentation by both major histocompatibility complex (MHC) class I and II molecules. Progress has been achieved in the preclinical research of HIV-1 VLPs as prophylactic vaccines that induce broadly neutralizing antibodies and potent T cell responses. Moreover, the progress in HIV-1 dendritic cells (DC)-based immunotherapy provides us with a new vision for HIV-1 vaccine development. In this review, we describe updates from the past 5 years on the development of HIV-1 VLPs as a vaccine candidate and on the combined use of HIV particles with HIV-1 DC-based immunotherapy as efficient prophylactic and therapeutic vaccination strategies.

## 1. Introduction

More than 30 years have passed since the discovery of the human immunodeficiency virus (HIV) in 1983 [1,2], and the development of an effective, safe and affordable HIV vaccine is still the first priority for achieving ultimate control of the worldwide HIV pandemic. Since 1987, more than 30 vaccine candidates have been tested in over 230 clinical trials [3,4], with only a few of these trials reaching phase IIb/III [5]. None of these candidates showed efficacy, except for the RV144 vaccine trial carried out in Thailand that reported an estimated efficacy of 31.2% [5,6]. The RV144 trial shed light on eliciting broadly neutralizing antibodies and structural-based vaccine design for developing an effective HIV-1 vaccine.

Several types of traditional vaccine strategies, such as live attenuated virus, inactivated virus and recombinant viral vectors, have been disappointing for the development of HIV vaccines. Live attenuated virus vaccines have been a great success in preventing small pox, measles and poliovirus infections, but this strategy has not been adopted for an HIV vaccine due to safety concerns, as live attenuated HIV may revert to a virulent pathogenic form, and the virus genome may integrate into the recipient’s genome [7,8]. Inactivated virus vaccines have been proven successful for controlling the prevalence of influenza and poliovirus worldwide. Although inactivated HIV has long been considered as a vaccine candidate, especially for therapeutic vaccines proposed by Jonas Salk in 1987 [9], it has been shown that an inactivated virus vaccine is a disappointing approach for HIV vaccine development. The traditional inactivation method such as formaldehyde treatment causes a loss of antigenicity, and virus inactivated using alternative inactivation approaches induced only modest neutralization [10]. It has been demonstrated that protection against SIV observed in macaques immunized with inactivated SIV was due to presence of host HLA-I antigen residue instead of SIV-specific immune responses [11,12,13]. Additionally, bio-safety issues in production and the risk of incomplete inactivation of HIV are also disadvantages of an inactivated HIV vaccine. Several recombinant viral vector and DNA vaccine candidates tested in clinical trials showed poor immunogenicity or no efficacy [14,15,16,17].

Recent efforts in HIV vaccine development have aimed at the induction of broadly neutralizing antibody and T cell responses. HIV-1 virus-like particles (VLPs) are highly attractive vaccine candidates due to their capability of presenting native functional Env spikes on the surface, which are able to elicit neutralizing antibodies. Additionally, HIV-1 VLPs can be captured by DCs and presented by both MHC class I and MHC class II molecules without leading to infection or replication [18,19,20]. VLPs are self-assembling, non-infectious, non-replicating genome-less virions that retain their natural structure and conformation and display authentic antigen [21,22,23,24,25]. Due to their particulate structure, VLPs can be taken up efficiently by APCs, especially DCs, leading to the induction of strong cellular and humoral immune responses [18,26,27,28,29,30,31]. At present, there are four VLP-based licensed vaccines in the market such as HBsAg VLPs (Engerix-B® by GlaxoSmithKline, Recombivac HB® by Merck and GenHevacB® by Sanofi-Pasteur, *etc.*), HPV VLPs (Cervarix® by GlaxoSmithKline, Gardasil® by Merck), HEV VLPs (Hecolin® by Xiamen Innovax Biotech) as well as Influenza virus VLPs (Flublok® by Protein Sciences Corporation), and a number of VLP-based vaccine candidates in clinical development [24,32].

Moreover, a growing endeavor has been focused on the development of therapeutic HIV-1 vaccines based on dendritic cells (DCs), which are able to restore T cell responses, limit virus replication and are financially more affordable as an alternative to life-long antiretroviral therapy (ART). DCs are the most potent professional antigen-presenting cells that play a pivotal role in shaping the magnitude and duration of immune responses [33,34,35,36]. DC-based therapy has been broadly tested for anti-tumor and anti-viral therapy [37,38,39,40,41]. In 2010, the US FDA approved the first DC-based cancer vaccine [42]. DCs play a central role in HIV infection. It is expected that DC-based vaccine approaches will induce strong CD4+ T cell responses that are critical for a sustained and effective HIV-specific CD8+ CTL response to suppress HIV replication, while DCs also contribute to virus dissemination.

We briefly review the current progress in the development of the HIV-1 VLP vaccine and DC-based HIV-1 immunotherapy as well as the features of these two immunization strategies and, we also emphasize that the combination of these two immunization strategies may be a prophylactic and/or a therapeutic vaccination approach against HIV-1 infection.

## 2. Current Progress in HIV-1 VLP as a Prophylactic Vaccine Candidate

### 2.1. HIV-1 Envelope Glycoprotein in VLPs as an Immunogens

For an effective prophylactic HIV-1 vaccine, the capability of eliciting broadly neutralizing antibodies (bNAbs) that can inhibit the infection of diverse HIV strains is one of essential elements [16,43]. bNAbs bind to the native envelope glycoprotein spikes on the surface of HIV-1 and interfere with receptor engagement and virus entry into the cell [43,44]. Over the past 20 years, varieties of bNAbs have been isolated from naturally infected donors whose sera has shown potent cross-strain HIV-1 neutralization, and Env epitopes targeted by bNAbs have been identified. b12 and 2G12 directly recognize the exterior portion of the gp120 protein [45,46,47,48], while 2F5, 4E10 and Z13 target the transmembrane gp41 protein [49,50,51]. Recent studies demonstrated the blockade of the CD4 binding site by VRC01/2, VRC03 and HJ16 [52,53], the binding of PG9 and PG16 to discontinuous epitopes located in the trimeric structures [54], the neutralizing activities of a mAb against the V3 loop [55,56,57]. It is also noteworthy that more and more new bNAbs were described in the most recent years, such as the recognition of a glycan-dependent epitope at the interface of gp41 and gp120 of properly formed and cleaved trimmers by PGT151 [58,59], PGT121 blocking V3 loop of gp120 [60,61] and binding to a conformational but yet to be defined epitope by 3BC176 [62,63]. Therefore, Env has been considered as the principal antigen for HIV-1 VLP vaccine development, and great effort has been made to improve its ability for priming bNAb responses.

However, the poor accessibility to the neutralizing epitopes strongly inhibits the development of bNAbs, due to a number of features of Env. Heavy glycosylation results in the formation of a glycan shield on the surface of Env trimer, which reduces access to the protein epitopes [64,65,66,67]. Moreover, conserved neutralizing sites, such as epitopes in the CD4-binding site and the membrane-proximal external region (MPER), hide within the trimer. The CD4-binding site is not fully exposed and undergoes a conformational change upon its binding to CD4 [67]. Similarly, the exposure of MPER is coupled to the fusion between the virus and host cell membranes [68,69]. In addition, the low density of native Env spikes displayed on the viral surface, as well as the contamination with non-functional Env, also contribute to the inaccessibility of neutralizing epitopes [70,71,72,73,74,75].

To elicit a high titer of anti-Env neutralizing antibodies, enhancing the exposure of the neutralizing epitopes in Env on the surface of the VLPs could be an effective strategy. The immunization of rabbits with VLP displaying Env trimers showed that glycan-deficient patches allowed the development of tier 2 neutralizing antibodies to native spikes [76]. Elimination of a glycosylation site in Loop D and two glycosylation sites in variable region 5 of Env enabled Env to bind to and to activate B cells expressing germline-reverted BCRs of two potent VRC01 class broadly neutralizing antibodies [77,78]. Therefore, the absence of glycan facilitates the exposure of the neutralizing epitopes in Env, indicating that removing glycosylation sites in highly conserved epitopes of native Env could be an effective pathway to encourage bNAb development and to reinforce the potency of Env-bearing VLP candidates. Additionally, the direct presentation of conserved epitopes of Env on the surface of VLPs, avoiding the conformational masking of the neutralizing sites, may facilitate the induction of the bNAb response. A recent study showed that chimeric VLPs, a bovine papillomavirus (BPV) displaying gp41, presented MPER and induced 2F5 and 4E10 epitope-specific antibodies in mice, resulting in partial neutralization of clade B and clade C virus [79]. Another study showed that gp41 peptide arrays were presented at a high density on the surface of bacteriophage VLPs designated AC205 and induced cross-strain neutralizing antibodies to different strains of HIV-1 pseudovirus at various levels following immunization in mice [80].

It is widely accepted that the native Env trimer, rather than the non-functional forms of Env such as the uncleaved gp160 and gp41 stumps, elicited neutralizing responses [70]. HIV-1 presents approximately 14 Env trimers at a Gag:Env ratio of 60:1 on the surface of a native virion with an irregular distribution pattern [74,75,81]. The elimination of aberrant Env could be helpful to reduce non-neutralizing antibody development. “Trimer” VLPs bearing pure native Env spikes were generated by enzyme digestion to remove antigenic interference of non-functional Env, and the data showed that the trimer VLPs induced tier 2 neutralizing antibodies [76,82]. Because the low density of Env spikes on the VLP surface may negatively affect affinity selection [73,81], a high efficiency of HIV-1 envelope protein expression and presentation on the surface of the VLPs could benefit the induction of anti-Env NAbs. For this purpose, VLPs expressing recombinant HIV-1 Env with trimeric conformation was produced in insect cells using a baculovirus expression system, and sera from immunized mice showed a high reactivity with HIV specific antigens but a disappointing neutralization activity to HIV pseudovirus [83].

### 2.2. HIV-1 Gag Proteins in VLPs as Immunogens

Gag polyprotein is an essential component of HIV-1 VLPs that is capable of self-assembly, giving rise to VLPs without other viral proteins or virus RNA, which provides a platform for the presentation of envelope antigens [84,85,86,87]. Moreover, HIV-1 Gag has been considered as an attractive HIV antigen. In 2000, it was reported that HIV-1 p55^gag^ VLPs were capable of priming strong long-lasting CTL responses against multiple HIV-1 p55^gag^ epitopes in rhesus macaques [31]. A previous study showed that yeast-expressed HIV-1 p55^gag^ VLPs were efficiently incorporated into DCs, and VLP-loaded DCs activated Gag-specific CD8+ T cells, inducing a CTL response in chronically HIV-infected patients [30]. It has been reported that HIV-1 Pr55^gag^ VLPs induced HIV-1-specific CD4+ and CD8+ T cell responses, as well as antibody response with cross-clade but partial neutralizing reactivity to primary field isolate in BALB/C mice [88]. Recent studies demonstrated that Gag-VLP efficiently induced the activation and maturation of DCs and the production of pro-inflammatory cytokines, which subsequently mediated NK immune responses [89]. Moreover, the same research group also reported that HIV-1 Gag-VLP up-regulated the expression of APOBEC3G and 3F in an IFN-α-dependent manner in human DCs and inhibited HIV-1 replication in both DCs and CD4+ T cells [90]. These findings revealed that in addition to a platform for displaying Env antigen, HIV-1 Gag is also a promising HIV-1 vaccine component for prophylactic and therapeutic purposes.

### 2.3. Combinations of HIV-1 Proteins in VLPs as Immunogens

Although native Env trimers are used as the principal antigen for HIV VLP vaccines, suggestive evidence for a cross-talk between different lentiviral proteins in immune responses has also been observed. Several independent studies in which non-human primates immunized with SIV Gag and followed by SIV or SHIV challenge showed an elevated Env-specific or neutralizing antibody response after virus challenge in Gag-immunized macaques [91,92,93]. One study demonstrated that the anti-Env antibody increased significantly in mice immunized with an adenoviral vector or a DNA vaccine against Gag-Pol and boosted with a VLP bearing Gag-Pol and Env [94]. In addition, in the same study, an enhancement of the Env antibody response to VLP immunization in recipient mice was observed after the adoptive transfer of CD4+ T cells from Gag-Pol-immunized mice. Although the mechanism of the cooperation between the immune response to Env and that to Gag-Pol remains to be investigated, it has been suggested that “intrastructural help” could be one explanation, according to which it was speculated that whole viral particle may be taken up by naïve B cells and peptides of both Env and Gag may be presented on the MHC-II molecules of B cells, therefore Gag-specific T helper cells could provide cognate help for Env-specific B cells and thus facilitate Env-specific antibody responses [94]. This information reminds us that besides HIV-1 Env glycoprotein presented on the surface of VLPs. Other components of HIV-1 VLPs play a synergistic role that contribute to a more profound protective antibody responses. In a recent study, two attenuated poxvirus vectors expressing soluble gp140 and Gag-Pol-Nef as VLPs were employed as HIV-1 vaccine candidates and tested in mice, in which highly polyfunctional Env-specific CD4 responses, in the case of gp140, and high magnitude Gag-specific CD8 T cell responses, in the case of Gag-Pol-Nef, were elicited, respectively, and antibody responses against gp140 and p17/24 were observed as well [95]. Additionally, this study indicated that the combination of these two vectors expressing different HIV-1 antigens led to more balanced CD4 and CD8 T cell responses and better humoral and cellular immune responses. Therefore, optimization of the antigen composition pattern could be an efficient pathway to improve the immunogenicity and potency of HIV-1 VLP vaccines. In addition, these observations indicate the advantage of using VLPs as a vaccine approach.

### 2.4. Incorporation of Different Adjuvants into HIV-1 VLP to Enhance Immune Responses

In a series of recent studies, the adjuvant effects of different biological agents were tested to improve the immunogenicity and potency of HIV-1 VLPs.

Studies have shown that cytokines may play a role as effective adjuvants to enhance immune responses [96,97,98,99]. Anti-Env antibodies with strengthened avidities were induced in rhesus macaques vaccinated by a heterologous prime-boost regimen with a co-expression of GM-CSF and antigens [100]. It has been indicated that GM-CSF could promote the avidity of the antibody by recruiting and activating antigen-presenting cells (APCs), such as myeloid dendritic cells, which are important to elicit robust immune responses [100,101]. It has been demonstrated that IL-4 played a critical role in the regulation of Th2 lineage differentiation and thus benefited humoral immune responses [102]. Previous studies have shown that the combination of GM-CSF and IL-4 induces the differentiation and optimization of growth of DCs [103,104,105]. GIFT4 is a fusion protein of GM-CSF and IL-4, which has been shown to lead to an alteration in the pro-immune cytokine secretory profile and a robust B cell mitogenic response [106]. A recent study showed that GIFT4 was successfully anchored into HIV-like particles presenting a high density of Env as a co-stimulatory factor, forming a novel adjuvanted chimeric VLP as a HIV VLP vaccine candidate [107]. The immunogenicity of this novel chimeric HIV-1 VLP was evaluated in guinea pigs via an intramuscular priming-intranasal boosting route, and results showed that improved systematic and mucosal antibody responses with augmented avidities and broadened neutralizing breadth were generated, although only partial neutralization was achieved, compared to un-adjuvanted HIV-1 VLPs, neutralizing reactivity to certain strains of clade B and clade C virus was significantly enhanced. This research demonstrated the potential of GIFT4-containing HIV VLPs as a promising HIV vaccine candidate, as well as the positive role that cytokines could play as adjuvants enhancing the immunogenicity of HIV-1 VLPs.

Flagellin was tested as an adjuvant incorporated into HIV-1 VLPs in a guinea pig model [108]. Flagellin is the principal protein that forms the highly complex flagellar structures named flagellum that extend from the outer membrane of Gram-negative bacteria. A conserved site on flagellin can be recognized by Toll-like receptor 5, and it has been observed that flagellin is a potent and effective adjuvant in both humans and non-human primates [109,110,111,112]. It has been shown that HIV-1 VLPs with membrane-anchored flagellin induced a broadened neutralization activity against five strains from clades B and C, and the adjuvant effect of flagellin contributed to the enhancement of HIV-1 VLP immunogenicity.

Targeting antigens selectively to DCs has been proposed and studied as a novel approach to improve the effectiveness and potency of vaccines [113]. The CD40 ligand has been constructed into HIV-1 and SHIV VLPs successfully to target antigens to the CD40 receptors on DCs to enhance HIV-1-specific immune responses [114,115]. In these studies, the biological properties of these VLPs were characterized *in vitro* and immunogenicity was investigated in mice, which indicated that CD40L-containing VLPs could target to DCs and promote the activation of DCs and that CD4+ and CD8+ T cell responses were stimulated efficiently.

### 2.5. Mucosal Immunity against HIV-1 Induced by VLPs Bearing HIV-1 Antigen

HIV is predominantly transmitted venereally, with infection initiating at a mucosal surface such as the vaginal or rectal mucosa [116,117,118], and HIV pathogenesis studies have demonstrated that early infection at a mucosal portal of entry is a bottleneck for HIV infection [119]. Mucosal secretory IgA plays an essential role in preventing HIV infection at the mucosal portals of entry [120,121]. Therefore, various measures have been adopted to induce effective HIV-1-specific mucosal immunity. It has been shown that the nasal cavity is a critical site for induction of both mucosal and systemic immune responses, as intranasal immunization has been shown to be effective for preventing influenza infection [122,123,124,125,126,127,128]. Moreover, it has been reported that VLPs have shown a satisfactory track record in intranasal immunization due to their capabilities in efficiently targeting APCs and promoting the induction of potent immune responses [29,127,129,130,131,132,133,134,135]. The immunogenicity of chimeric HIV-1 VLP with flagellin anchored on the surface was tested in guinea pigs via intranasal immunization, where enhanced mucosal immunity was observed [108]. In another study, intranasal administration was adopted to test the immunogenicity of an HIV-1 VLP based on Gag protein consisting of whole HIV gp120/140 envelope protein derived from an Ugandan clade A field isolate in rhesus macaques, which showed that sequential intranasal and intramuscular administration of HIV VLPs, in contrast to intranasal administration alone, was able to elicit humoral immune responses at the systemic and the vaginal level, but suggested that intranasal administration may be priming the humoral mucosal immunity for subsequent intramuscular immunization [136]. Intranasal immunization has also been employed as a boosting immunization route. The immunogenicity of GIFT_4_-containing HIV-1 VLPs was tested in guinea pigs through an intramuscular priming-intranasal boosting route and mucosal immunity was evaluated by measuring the titer of IgG and IgA at mucosal sites, where an increased level of antibodies was observed, indicating that immunity at the mucosal site was successfully elicited [107]. It has been demonstrated that BPV VLP is an ideal mucosal delivery vector that protects antigens from degradation in the digestive tract and functions as an adjuvant, inducing both mucosal and systemic immune responses through the oral immunization route [137,138,139,140,141,142]. The oral immunization route was adopted to test the immunogenicity of a HIV-1 MPER domain displaying bovine papillomavirus (BPV)-based VLPs in mice, and HIV-specific systemic immune responses and mucosal immune responses at the intestinal mucosa were successfully induced, although a high titer of antibodies at the vaginal mucosa was not detected [79]. Therefore, various pieces of evidence have demonstrated that HIV VLPs are capable of eliciting mucosal immune responses, although protection at the mucosal site needs to be further validated by challenge with HIV-1, for example.

## 3. HIV-1 VLPs as Potential Antigens in DC-Based HIV-1 Therapeutic Vaccination

### 3.1. Current Progress of DC-Based HIV-1 Therapeutic Vaccine

A DC-based therapeutic vaccine is intended to restore HIV-1-specific T cell responses, control viral replication or even eradicate the HIV reservoir in HIV-infected individuals. A successful DC-based therapy shall be able to persistently suppress HIV replication efficiently without life-long cART, which is considered as a “functional cure”. A series of *in vitro* tests demonstrated that T cell immune responses can be induced by DCs loaded with various HIV antigens. It has been reported that monocyte-derived DCs pulsed with aldrithiol-2-inactivated autologous virus stimulated the proliferation of both CD4+ and CD8+ T cells in patients, as well as CTL responses of CD8+ T cells [143]. Subsequently, another two studies reported *ex vivo* autologous inactivated HIV-1 production for a clinical DC-based therapeutic immunization, and both of these studies demonstrated that autologous inactivated HIV-1 virus-based immunogen production fulfilled cGMP and the expected specifications and could be used for the purpose of clinical trials [144,145]. DCs pulsed with HIV-1-derived proteins have been studied. DCs pulsed with HIV-1 Gagp55-liposome complexes have been shown to be capable of stimulating PBMCs from HIV-1-negtive individuals and inducing HIV-1-specific CD8+ T cell responses [146]. DCs loaded with synthetic peptides representing HIV-1-specific HLA class I epitopes activated blood-derived autologous CD8+ T cells [147]. HIV-1 antigen-encoding mRNA electroporation has been demonstrated to be a more effective technique, and HIV-1-specific CD4+ and CD8+ T cell responses were stimulated by DCs from both infected and uninfected individuals electroporated with mRNA encoding Gag p24 and Gag p55, Rev and Nef, and Env, Nef, as well as Tat [148,149,150,151,152,153,154]. Additionally, studies have demonstrated the T cell activation capabilities of DCs pulsed with HIV-1 antigen expressed through recombinant viral vectors and HIV-1 antigen-displaying nanoparticles [155,156,157,158,159].

Potent immune responses against HIV-1 have been demonstrated by animal studies in which animals were immunized with DCs bearing inactivated virus, HIV-1 viral lysate or envelope glycoproteins [160,161,162,163]. To date, 17 clinical trials of DC-based HIV-1 immunotherapy have been reported, as shown in Table 1 [154,164,165,166,167,168,169,170,171,172,173,174,175,176,177,178,179]. In these clinical trials, DCs were loaded with a variety of types of HIV-1 antigens, including AT-2- or heat-inactivated HIV-1, HIV-1 peptides, HIV-1 antigen-encoding RNA or HIV-1 antigen-expressing recombinant viral vectors. All of these clinical trials demonstrated an excellent safety profile of the DC-based HIV-1 therapy, with only minor local side effects as reported in some clinical trials. No severe side effects or induction of autoimmunity were reported. Different levels of virological responses to immunization were observed. Studies performed with autologous antigens showed variable levels of virological responses [165,166,171,172,175], which correlated with consistent enhanced HIV-1-specific T cell immune responses, and clinical trials performed with heterologous antigens failed to show any virological responses to immunization, although moderate to strong immune responses were observed in these studies, such as increased CD4+ T cell count, HIV-1 antigen-specific CTL responses and IFN-γ production [164,167,168,169,170,176]. All of the 17 clinical trials suggested that DC-based immunotherapy in HIV-1 infection is able to induce HIV-1-specific immunological responses, including both HIV-1-specific CD4+ and CD8+ T cell responses. Although prominent variability existed in all aspects of these clinical trials, including the choice of HIV-1 antigens, preparation of DC, design of clinical trial and immunological assessment, the results of these clinical trials are promising and inspiring, providing us with clues for the further development of DC-based HIV-1 therapy or HIV-1 vaccination strategy.

**Table 1:** Reported clinical trials of dendritic cell (DC)-based HIV-1 therapeutic vaccine.

Author [Ref.]	HIV-1 Antigens	DC Type	DC Maturation	Subjects	Treatment	Loading Strategy	Route	Schedule
Kundu *et al.* [164]	Recombinant HIV-1 gp160 or synthetic env, gag, pol peptides	allogeneic or autologous blood DC	-	N = 6	-	Pulsing	I.V.	6–9 doses at monthly intervals
Lu *et al.* [165]	AT2 inactivated autologous virus	autologous MDDC	IL-1β, IL-6, TNF-α	N = 18	-	Pulsing	S.C.	3 doses every 2 weeks
Garcia *et al.* [166]	heat inactivated autologous virus	autologous MDDC	IFN- α	N = 18	HAART	Pulsing	S.C.	4 doses every 6 weeks
Ide *et al.* [167]	synthetic peptides with HLA-A*2402 restriction of Gag, Pol, Env	autologous MDDC	TNF-α	N = 4	HAART	Pulsing	S.C.	6 doses every 2 weeks
Connolly *et al.* [168]	synthetic peptides with HLA-A*2402 restriction of Gag, Pol, Env	autologous MDDC	IL-1β, IL-6, TNF-α	N = 18	ART	Pulsing	I.V.&S.C.	2 doses with 3 week interval
Gandhi *et al.* [169]	ALVAC-HIV-1 vCP1452 (Sanofi-Pasteur)	autologous MDDC	Monocyte-conditioned medium containing IL-1β, IL-6, TNF-α, PEG2	N = 29	ART	DCs infected with live virus	S.C.	3 doses at week 3, 7, 15
Kloverpris *et al.* [170]	synthetic peptides with HLA-A*0201 restriction of Gag, Vif, Env, Vpu, Pol	autologous MDDC	IL-1β, IL-6, TNF-α, PEG2	N = 12	-	Pulsing	S.C.	4 doses at week 0, 2, 4, 8
Routy *et al.* [154]	RNA encoding Gag, Rev, Vpr, Nef & CD40L	autologous MDDC	TNF-α, IFN-γ, PEG2	N = 10	ART	Electroporation	I.D.	4 doses at week 0, 4, 8, 12
Gracia *et al.* [171]	heat inactivated autologous virus	autologous MDDC	IL-1β, IL-6, TNF-α	N = 24	-	Pulsing	S.C.	3 doses every 2 weeks
J.P.Routy *et al.* [172]	RNA encoding Gag, Rev, Vpr, Nef & CD40L	autologous MDDC	TNF-α, IFN-γ, PEG2	N = 29	ART	Electroporation	S.C.	4 doses at week 0, 4, 8, 12 and 2 booster doses after ART interruption
Allard *et al.* [173]	RNA encoding Tat, Rev, Nef	autologous MDDC	GM-CSF, IL-4, IL-1β, IL-6, TNF-α, PEG2	N = 17	cART	Electroporation	S.C.&I.D.	4 doses at week 0, 4, 8, 12
Van Gulck *et al.* [174]	RNA encoding Gag & chimeric Tat-Rev-Nef protein	autologous MDDC	TNF-α, PEG2	N = 6	HAART	Electroporation	I.D.&S.C.	4 doses at week 0, 4, 8, 12
Garcia *et al.* [175]	heat inactivated autologous virus	autologous MDDC	IL-1 β, IL-6, TNF-α, PEG2	N = 36	cART	Pulsing	S.C. or I.D.	3 doses at week -4, -2, 0 & week 0, 2, 4 respectively
Levy *et al.* [176]	ANRS HIV LIPO5 peptides: gag, pol, nef	autologous MDDC	LPS	N = 19	HAART	Plusing	S.C.	4 doses at week 0, 4, 8, 12
Tcherepanova *et al.* [177]	RNA encoding Gag, Rev, Vpr, Nef	autologous MDDC	Unkown	N = 36	ART	Electroporation	Unknown	4 doses at week 0, 4, 8, 12
Gandhi *et al.* [178]	RNA encoding Gag, Nef	autologous MDDC	IL-1β, IL-6, TNF-α, PEG2	N = 15	cART	Electroporation	I.D.	4 doses at week 0, 2, 6, 10
Macatangay *et al.* [179]	Autologous inactivated HIV-1-infected apoptotic cells	autologous MDDC	TNF-α, IFN- α IFN-γ, IL-1β, Poly(I:C)	N = 10	ART	Pulsing	S.C.	3 doses at week 0, 2, 4,& the 4th dose at week 24

MDDC: Monocyte-derived dendritic cells; S.C.: subcutaneous injection; I.D.: Intradermal injection; I.V.: intravenous injection; HAART: Highly active antiretroviral therapy ; ART: antiretroviral therapy; cART: combination antiretroviral therapy.

### 3.2. Potential Role of HIV-1 VLP in DC-Based HIV-1 Immunization

Although encouraging results of DC-based HIV-1 immunotherapy clinical trials have been reported, a functional cure has not been achieved in any patient yet. There are several possibilities for improving the therapeutic effect of DC-based vaccines, including a novel form of HIV-1 antigen for DC pulsing, an improved DC pulsing method that enhances antigen presentation, different adjuvants that facilitate immune responses and optimization of clinical trial design, which will help in the data analysis and assessment of the efficacy of the vaccines. Regardless of the other factors that may affect the outcomes of the 17 clinical trials, the types of HIV-1 antigens utilized in these 17 clinical trials showed different degree of therapeutic effects, especially the decrease of viral load.

A previous clinical trial using DCs loaded with an AT-2-inactivated autologous virus showed a sustained viral load drop [165], and it was indicated that AT-2 inactivation preserves the conformational and functional integrity of the virion surface proteins, which may facilitate DC antigen presentation to induce potent CD4+ and CD8+ responses [180,181,182]. The HIV-1 VLP consists of viral capsid proteins that self-assemble into particulate structures and displays native functional Env spike on the surface, imitating the natural virus particles. Because HIV-1 VLP is not infectious, there is no need for inactivation. Based on the features of HIV-1 VLP, we would like to hypothesize that HIV-1 VLP may play a role as antigen for DC-based therapy and that the native Env trimers displayed on the VLP may also facilitate the development of broadly neutralizing antibody responses, which are beneficial for the control of viral load and clinical of progression of HIV infection.

In addition, the DCs loaded with HIV-1 VLP would be a supplementary boosting regimen for HIV-1 VLP vaccination. Administration of DCs loaded with HIV-1 antigen could be the most direct way of presenting antigens, and clinical trials of DC-based HIV-1 therapy have shown that T cell responses were successfully induced in HIV-1-infected patients with compromised immunity. Studies have demonstrated that a potent HIV-1-specific CD4+ T cell response is critical to induce a sustained and efficient HIV-specific CD8+ T response, which is important in controlling HIV replication by killing HIV-1-infected cells [183,184,185,186,187]. Signals provided by CD4+ T cells are also essential for memory B cell differentiation and affinity maturation [188]. Thus, an effectively and properly primed CD4+ T cell response is fundamental for the development of protective anti-HIV-1 immune responses. DCs are potent professional APCs and are crucial for the induction of HIV-1-specific CD4+ T cell responses. Efficient antigen presentation benefits the priming of HIV-1 CD4+ T cell responses. Moreover, it is reported that HIV Gag-Pol-specific CD4+ T cells were capable of providing intrastructural help for Env-specific B cells and therefore facilitate antibody responses against HIV-1 [94]. Therefore, it is reasonable to assume that DC-based HIV immunization could be an effective way to induce immune responses to HIV-1 in healthy individuals. However, due to the complexity of DC-based immunization, we would like to propose DCs loaded with HIV-1 VLP as a supplementary boosting regimen for HIV-1 VLP vaccination.

## 4. Conclusions

HIV-1 VLP is a promising vaccine consisting of a viral capsid protein and native Env trimers presented on the surface, which could induce host immunological responses against HIV infection. Various modifications of VLPs can be adopted to improve the immunogenicity of HIV-1 VLPs. DC-based HIV therapeutic treatment has been explored as an alternative treatment of ART, which exhibited an excellent safety profile, and clinical trials demonstrated anti-HIV-1 immune responses induced by DC-based immunization, although a functional cure has not been achieved yet. Considering the advantages of HIV VLPs and DC-based HIV-1 treatment, we would like to suggest combining the two immunization strategies so that HIV-1 VLPs could be a potential antigen for DC-based HIV-1 therapeutic treatment, and this VLP-mediated DC-based vaccination could be an effective method for both mucosal and systematic immunization to boost the host immune responses against the HIV-1 pandemic.
